# ﻿*Endoxocrinus* (*﻿Diplocrinus*) *﻿kexuei*, a new species of stalked crinoid (Echinodermata, Crinoidea, Isocrinida, Balanocrinidae) from rotten wood in the cold seep area of the Taixinan Basin, South China Sea

**DOI:** 10.3897/zookeys.1241.128991

**Published:** 2025-06-12

**Authors:** Shao’e Sun, Zijie Mei, Zhongli Sha

**Affiliations:** 1 Department of Marine Organism Taxonomy & Phylogeny, Institute of Oceanology, Chinese Academy of Sciences, Qingdao 266071, China; 2 Laboratory for Marine Biology and Biotechnology, Qingdao Marine Science and Technology Center, Qingdao 266237, China; 3 Shandong Province Key Laboratory of Experimental Marine Biology, Institute of Oceanology, Chinese Academy of Sciences, Qingdao 266071, China; 4 University of Chinese Academy of Sciences, Beijing 100049, China

**Keywords:** Cold seeps, *Endoxocrinus*, new species, South China Sea, stalked crinoids, systematics

## Abstract

A new species of stalked crinoid, Endoxocrinus (Diplocrinus) kexuei**sp. nov.** belonging to the family Balanocrinidae, is described from cold seeps in the South China Sea. The general appearance of the new species is similar to E. (Diplocrinus) alternicirrus, but can be distinguished from its congener in both morphological characteristics and significant genetic divergences. Endoxocrinus (Diplocrinus) kexuei**sp. nov.** shows cryptosymplexies without marked symmorphy, and an axial canal usually incompletely filled with a lattice needlelike network, preserving an irregular secondary lumen. The new species attribution is well supported by genetic distance based on the mitochondrial *c* oxidase subunit I (*COI*), and molecular phylogenetic analyses based on *COI* and *16S* rRNA. This discovery enhances our understanding of species diversity of *Endoxocrinus* crinoids in the South China Sea.

## ﻿Introduction

The order Isocrinida Sieverts-Doreck, 1952 comprises species that are among the most prevalent post-Paleozoic stalked crinoids, found both in the fossil record and among living crinoids (Améziane et al. 2023). The extant Isocrinida diverged into two major lineages during the Middle Triassic, generally recognized as two distinct families: Isocrinidae Gislén, 1924 (including Isocrininae and Metacrininae) and Balanocrinidae Roux, 1981 (including Balanocrininae, Diplocrininae, Isselicrininae, and Proisocrininae) (Améziane et al. 2023). They exhibit a heteromorphic pentaradiate stalk with nodals that carry cirri, and a flexible pinnulate crown typically possessing ten or more arms (David et al. 2006). The subfamily Diplocrininae, which separated from Balanocrininae during the Early Cretaceous, is distinguished by a notable shortening of the brachitaxes, the widespread replacement of axial synarthries in the arms with synostoses, and the consistent presence of paedomorphic symplexies in the stalk (Roux et al. 2009; Améziane et al. 2023). Diplocrininae is primarily found in the western tropical and northeastern Atlantic, the northern Indian Ocean, and the western and central Pacific, typically inhabiting depths ranging from about 200 to 2000 meters. (David et al. 2006). They grow on slopes and seamounts, thriving on both unconsolidated and rocky substrates (Améziane and Roux 1997; David 1998; David et al. 2006). Two extant genera, *Teliocrinus* Döderlein, 1912 and *Endoxocrinus* A.H. Clark, 1908, are currently attributed to Diplocrininae.

The main characters of the genus *Endoxocrinus* are: (1) IBr2ax, (2) arm branching usually endotomous, (3) B_r1+2_ of each brachitaxis connected by synostosis with small patches of syzygial stereom, and (4) mature symplexies and cryptosymplexies without an interpetaloid groove (David et al. 2006). The genus *Endoxocrinus* is subdivided into two subgenera, E. (Endoxocrinus) A.H. Clark, 1908 and E. (Diplocrinus) Döderlein, 1912. The sole species in the subgenus E. (Endoxocrinus) has more brachitaxis; the number of internodals in each mature noditaxis ranges from 3 to 16; a distal callus at the end of the stalk is rarely present; and the number of cirrals per cirrus varies from 25 to 43, with the mode typically exceeding 30, and the proximal cirri are either perpendicular to the stem or pointing downward (David et al. 2006). Endoxocrinus (Diplocrinus) spp., usually have two brachials in all brachitaxes; the number of internodals per mature noditaxis is strongly variable; usually with a callus on the distal facet of the distalmost nodal, and with proximal cirri pointing in variable directions (David et al. 2006). The subgenus E. (Endoxocrinus) is monospecific with E. (Endoxocrinus) parrae (Gervais in Guérin, 1835); whereas Endoxocrinus (Diplocrinus) includes three species, E. (Diplocrinus) alternicirrus (Carpenter, 1882), E. (Diplocrinus) maclearanus (Thomson, 1878), and E. (Diplocrinus) wyvillethomsoni (Thomson, 1872).

David et al. (2006) conducted a revision of the stalked crinoid species attributed to *Endoxocrinus*, in which ecophenotypic variations have been documented, highlighting the differences between adaptive and phylogenetically indicative traits. David et al. (2006) identified three ecophenotypes of E. (Endoxocrinus) parrae (Gervais in Guérin, 1835) assigned as subspecies: E. (Endoxocrinus) parrae parrae (Gervais in Guérin, 1835), E. (Endoxocrinus) parrae prionodes H. L. Clark, 1941 and E. (Endoxocrinus) parrae carolinae (A. H. Clark, 1934). Similarly, E. (Diplocrinus) alternicirrus includes three subspecies adapted to different habitats: E. (Diplocrinus) alternicirrus (Carpenter, 1882) and E. (Diplocrinus) sibogae (Döderlein, 1907).

Cold seeps are inhabited by a chemosynthetic community that utilizes released reduced compounds, primarily methane and hydrogen sulfide (Kato 2019). Compared to mollusks and arthropods, echinoderms are extremely rare in cold seeps, and paleoecological information of fossil echinoderms in or near cold seep environments is also sporadic (Hunter et al. 2016; Kato 2019; Forel et al. 2024). Since the identification of the first cold seep system in the northern South China Sea in 2004, over 40 active and ancient cold seeps have been found in regions such as the Qiongdongnan Basin, Xisha area, Shenhu area, Dongsha area, and Taixinan Basin (Berndt et al. 2014; Feng et al. 2018; Zhang et al. 2021). Studies on the species diversity of crinoids in the cold seep regions of the South China Sea are underdeveloped. Crinoids are generally not reported in cold seeps (Kato et al. 2017). Currently, only a few fossil isocrinids have been discovered in cold seep areas worldwide. Hunter et al. (2016) found a new stalked crinoid (*Lakotacrinusbrezinai*) from cold methane seeps in the Upper Cretaceous (Campanian) Pierre Shale, South Dakota, United States. Kato (2019) discovered fossil Isocrinina crinoids in the Upper Cretaceous cold seep deposits of the Yezo Group, Hokkaido, Japan. Forel et al. (2024) found some crinoids from the deposits at Sahune, Drôme, south-eastern France Basin, Middle Oxfordian, Late Jurassic.

During our surveys (Institute of Oceanology, Chinese Academy of Sciences) of the deep-sea fauna in the Northwestern Pacific Ocean in 2016, two unusual specimens of *Endoxocrinus* were discovered in the cold seep in the Taixinan Basin, South China Sea. Morphological examination and molecular phylogenetic analysis suggested that these specimens differ from other species of *Endoxocrinus*. Here, we describe them as a new species belonging to the subgenus Diplocrinus in the genus *Endoxocrinus*: Endoxocrinus (Diplocrinus) kexuei sp. nov.

## ﻿Material and methods

### ﻿Sampling and preservation

We conducted a deep-sea biodiversity survey in the Northwestern Pacific Ocean on 8 September, 2016, where we collected two specimens from a rotten piece of wood in the cold seep area of the Taixinan Basin, South China Sea. The two specimens were collected with a manipulator by the remotely operated submersible (ROV) *FaXian* (Discovery in Chinese) at a depth of 833.7 m, station FX-Dive125 (22°02.58'N, 118°46.83'E). The two specimens were preserved in 70% ethanol and deposited at the Marine Biological Museum of the Chinese Academy of Sciences (MBMCAS), Institute of Oceanology, Chinese Academy of Sciences (IOCAS). Holotype: MBM287584; Paratype: MBM287585.

### ﻿Morphology observations

Linear architectural characteristics (≥1 mm) of preserved specimens were measured with digital vernier calipers. For the curvilinear structures which were difficult to measure, a ZEISS Axiocam 506 (Carl Zeiss AG, Oberkochen, Germany) microscope camera was used to take photographs, and the Leica LAS Image Analysis software was used to conduct the measurements. Line drawings were completed in Adobe Photoshop 2021 using a graphics tablet. Measurements were rounded to the nearest 0.1 mm. Ossicles of the stalk and arm were dissociated with a 10% aqueous sodium hypochlorite solution until soft tissue was digested. Then the ossicles were rinsed with cold distilled water and dried at room temperature. Scanning electron microscope (SEM) observations were conducted using a Hitachi S-3400N SEM at an accelerating voltage of 5 kV.

The previous revision of pentacrinid stalked crinoids of the genus *Endoxocrinus* (David et al. 2006) and several published keys for different modern stalked crinoid (Roux et al. 2002) were used for the identification of *Endoxocrinus* species. We refer the reader to David et al. (2006) for morphology of *Endoxocrinus* and Roux (1977) for articulations of extant isocrinids. The abbreviations used herein are listed in Table 1.

**Table 1.** Quantitative morphological characters and the abbreviations of the holotype (MBM287584) and paratype (MBM287585) of *Endoxocrinus* specimens from South China Sea attributed to E. (Diplocrinus) kexuei sp. nov. For abbreviations see text. Measurements in mm. *indicate measurements done on the figure instead of the animal. ^a^ measurement based on remaining stalk length of paratype.

Abbreviations	Diagnostic characters	Holotype	Paratype
dp	proximal diameter of stalk	5	4.4
dd	distal diameter of stalk	4.3	3.7*
sL	stalk length	65	34.97*(17^a^)
InN	number of internodals per noditaxis	5	5*
nL	length of noditaxis	7.8	5.9*
InL	internodal length	1.4	0.8*
nL	nodal length	2.5	2.09*
cL	cirrus length	37.1	34.5*
cN	number of cirrals per cirrus	30	—
c_1_L	length of first cirral	0.9	—
c_1_W	width of first cirral	2.1	—
c_L_L	longest cirral length	1.74	—
c_L_W	longest cirral width	1.65	—
Arms	number of arms	22	20
bW	basal width	1.7	1.4
bL	basal length	1.1	1.4
rW	radial width	5.1	4.1
rL	radial length	1.8	1.2
p_1_L	length of first pinnule	10.8	8.1
p_1N_	number of pinnulars on first pinnule	11	9
p_1_L	first pinnular of first pinnule length	0.9	1
p_1_W	width of first pinnular of first pinnule	1.4	1.1

### ﻿DNA extraction, sequencing and phylogenetic analyses

Both *Endoxocrinus* specimens were prepared for DNA barcoding. All genomic DNA was obtained from pinnules using the E.Z.N.A.® Tissue DNA Kit (Omega-biotek, Inc., Norcross, Georgia, USA) according to the manufacturer’s instructions. The DNA was eluted using sterile distilled water and stored at −20 °C. Two mitochondrial markers, cytochrome *c* oxidase subunit I (COI) and large ribosomal RNA (16S rRNA), and one nuclear marker, *28S* ribosomal RNA (*28S* rRNA) were amplified by polymerase chain reaction (PCR), which was carried out in a reaction mix containing 1 μL of template DNA, 12 μL of Premix Taq™ (Takara, Otsu, Shiga, Japan), 1 μL of forward and reverse primers (10 mM), and 10 μL sterile distilled H_2_O to a total volume of 25 μL.

*COI*, *16S*, and *28S* were amplified using the primers and protocols outlined by Hemery et al. (2012). PCR products were examined by 1.5% agarose gel electrophoresis and purified using EZ-10 Spin Column DNA Gel Extraction Kit (Sangon Biotech, Shanghai, China) prior to sequencing. The purified PCR products were sequenced in both directions using an ABI PRISM 3730 (Applied Biosystems, Thermo Fisher Scientific, Massachusetts, U.S.A.) automated DNA sequencer, using the same primer sets as for PCR amplification.

Sequence data was visualised and edited by the Seqman software (DNASTAR, Inc., Madison, Wisconsin, USA). Manual checks ensured the accuracy of the sequences. These sequences were checked against the nucleotide NCBI database through BLAST searches (https://blast.ncbi.nlm.nih.gov/doc/blast-help/references.html#references) to ensure that the sequences were uncontaminated. New sequences were deposited on GenBank (Table 2).

**Table 2.** List of sequences produced or retrieved from NCBI used in the barcoding and phylogenetic analysis of extant isocrinids.

Taxon	*COI*	*16S*	*28S*	Reference
E. (Diplocrinus) kexuei sp. nov. (holotype)	OR077296	OR082608	PQ770959	Present paper
E. (Diplocrinus) kexuei sp. nov. (paratype)	OR077297	PQ770958	PQ770960	Present paper
E. (Diplocrinus) wyvillethomsoni	OR237781	OR233480	OR233473	Améziane et al. 2023
E. (Endoxocrinus) parrae	OR237782	GU327874	OR233474	Améziane et al. 2023
E. (Diplocrinus) alternicirrus alternicirrus	KC626541	KC626633	KC626821	Hemery et al. (2013)
E. (Diplocrinus) alternicirrus sibogae	KC626542	KC626634	KC626822	Hemery et al. (2013)
*Panglaocrinusisseliformis*	OR237780	OR233479	OR233472	Améziane et al. 2023
*Proisocrinusruberrimus*	GU327842	GU327878	GU327949	Rouse et al. (2013)

Two sequence datasets were analyzed. The first dataset included all existing *COI* barcoding sequences of *Endoxocrinus* and was used to perform species identification. The second dataset was used to determine the systematic status of new species of *Endoxocrinus*, *COI*, *16S* and *28S* concatenated sequences of seven *Endoxocrinus* taxa and an outgroup of *Panglaocrinus* (Table 2). For the protein-coding gene *COI*, alignment was performed with MEGA v.6 (Tamura et al. 2013) based on codon positions. The *16S* and *28S* sequences were instead aligned with default parameters using MAFFT v. 5 (Katoh et al. 2005), with G-INS-i strategy. Alignments were then trimmed using Gblocks 0.91 (Castresana 2000; Talavera and Castresana 2007) under default conditions. Kimura-2 parameter (K2P) genetic distances for *Endoxocrinus* were calculated using MEGA v. 6.

Phylogenetic trees were inferred using maximum likelihood (ML) and Bayesian inference (BI) analysis of tandem sequences. The most suitable partitioning scheme and replacement model were selected by PartitionFinder v. 2.1.1 (Lanfear et al. 2017) (*16S*, TrN+I+G; *COI*-1^st^ codon, HKY+I+G; *COI*-2^nd^ codon, SYM+I+G; *COI*-3^rd^ codon, GTR+G; *28S*, TPM2+F). ML analysis was performed using the IQ-TREE web server (Trifinopoulos et al. 2016), with best-fit partitioning scheme and models, selected with PartitionFinder v. 2.1.1. Branch support was assessed by 5000 ultra-fast bootstrap replications (Minh et al. 2013). BI analyses were performed with MrBayes 3.1 (Ronquist and Huelsenbeck 2003) using the partitioning model, the same used for IQ-TREE. A Markov chain Monte Carlo (MCMC) was run for 5,000,000 generations, sampling every 500 generations to allow sufficient time for convergence. The first 25% of the sampled trees were discarded as burn-in. The remaining trees were used to estimate the consensus tree with 50% majority rule and Bayesian posterior probability (PP). At the end of the run, the mean standard deviation of the split frequency was reduced to 0.01. The effective sample size (ESS) values for all sampling parameters were checked by Tracer v. 1.7 (Rambaut et al. 2018) to ensure that convergence was achieved.

## ﻿Results and discussion

### ﻿Systematics

**Order Isocrinida Sieverts-Doreck, 1952**

**Family Balanocrinidae Roux, 1981**

**Subfamily Diplocrininae Roux, 1981**

**Genus *Endoxocrinus* A.H. Clark, 1908**

#### Subgenus ﻿ Diplocrinus

Taxon classificationAnimaliaIsocrinidaBalanocrinidae

Döderlein, 1912

6BADD768-1834-548E-95A9-595588D4AF23

##### Type species.

*Pentacrinusmaclearanus* Thomson, 1872.

#### ﻿ Endoxocrinus (Diplocrinus) kexuei sp. nov.

Taxon classificationAnimaliaIsocrinidaBalanocrinidae

BEF603F2-8B96-54C4-B7C6-CC6AE6C89226

https://zoobank.org/7D41C791-0061-49C5-8CA1-9AA942E76F27

Figs 1 , 2 , 3 , 4 , 5 , 6 , 7 , 8 , 9

##### Material examined.

***Holotype*** • MBM287584, collected from one rotten piece of wood in the cold seep area of the Taixinan Basin, South China Sea, at the station FX-Dive 125 (22°02.58'N, 118°46.83'E), depth 833.7 m, hard substrate. ***Paratype*** • MBM287585 (one specimen), collection data same as the holotype.

##### Diagnosis.

Arm number 20–22; arms smooth, up to 14 cm long; lateral flanges present on all proximal brachials; IB_r1+2_ synostosis very flat with synostosial stereom predominating; axial canal rectangular; stalk shorter than arms, 6.5 cm long; internodals per mature noditaxis 5–8; stalk stellate to pentagonal cross-section; proximalmost diameter of stalk 5 mm; less than five (down to 2) cirri per nodal; proximal cirri directed upward; cirrus sockets large and round; 30 robust cirrals per cirrus; cirri rudimentary until 3^th^ nodal; cryptosymplexies without marked symmorphy and with facets mainly covered by synostosial stereom axial canal usually incompletely filled with stereom needlelike network and preserving an irregular secondary lumen

##### Etymology.

The species name is derived from the oceanographic vessel “Kexue” of the Institute of Oceanology, Chinese Academy of Sciences, which made a significant contribution to the biological research in the South China Sea.

##### Description.

**Figure 1.**
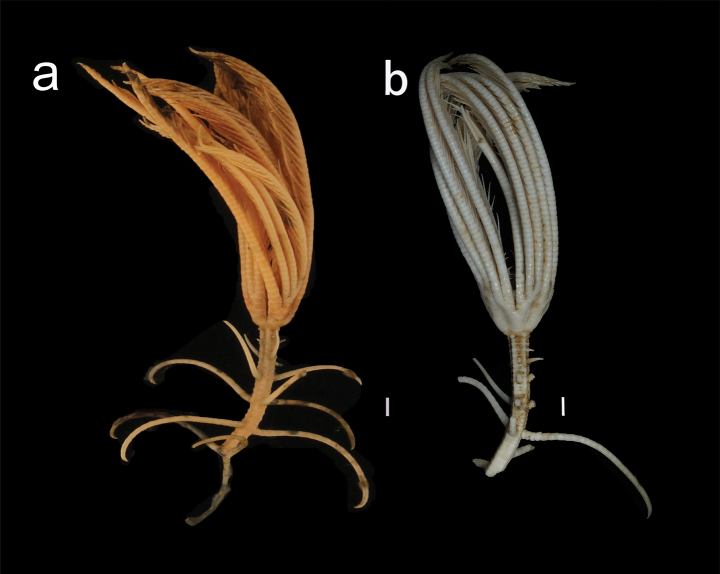
Endoxocrinus (Diplocrinus) kexuei sp. nov., holotype (MBM287584) **a** photograph of the entire organism taken directly after collection presenting the original colour **b** overall photo after preservation in alcohol. Scale bars: 1 cm.

**Figure 2.**
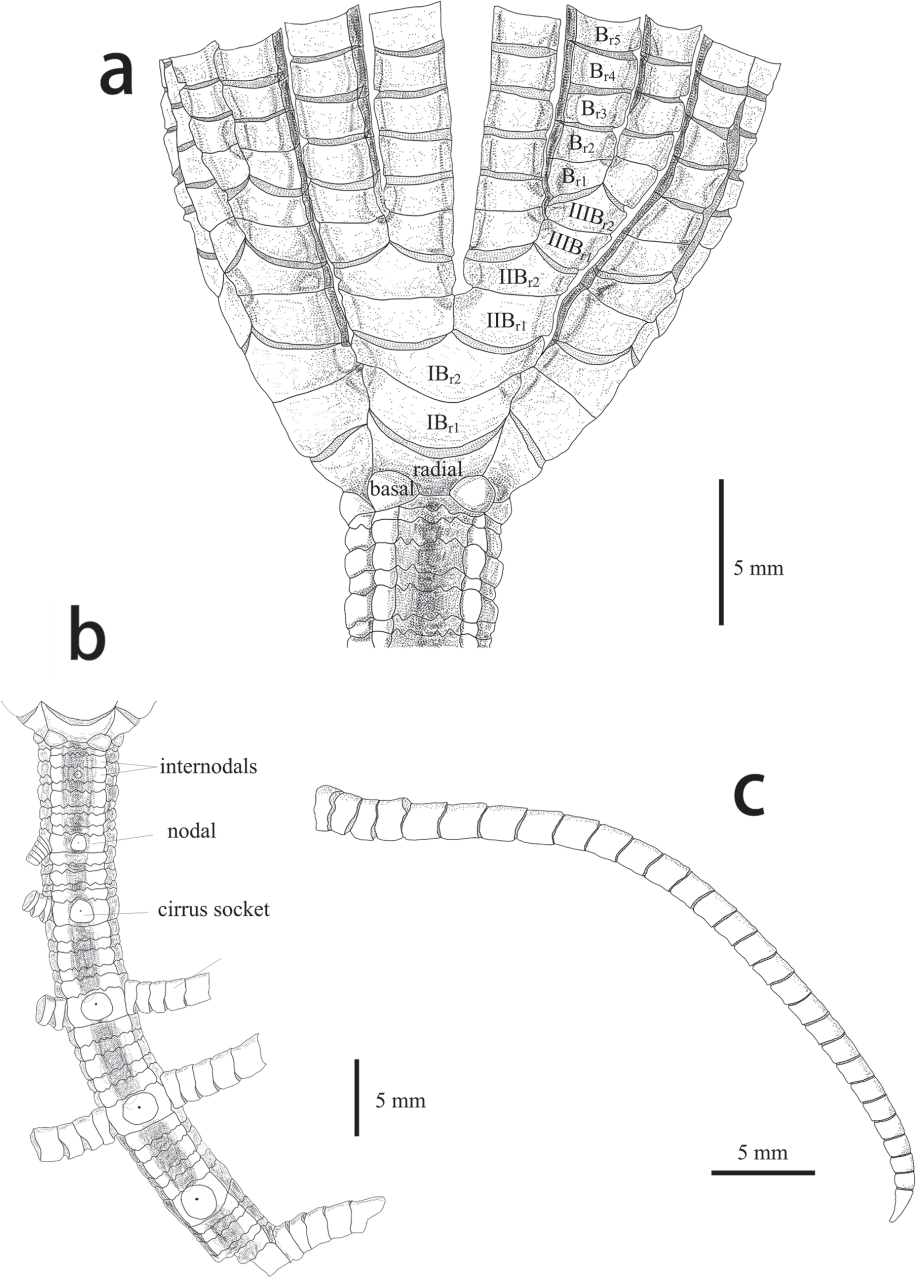
Endoxocrinus (Diplocrinus) kexuei sp. nov., holotype (MBM287584) **a** proximal stalk and base of crown **b** entire stalk **c** detail of complete cirrus. Scale bars: 5 mm.

**Figure 3.**
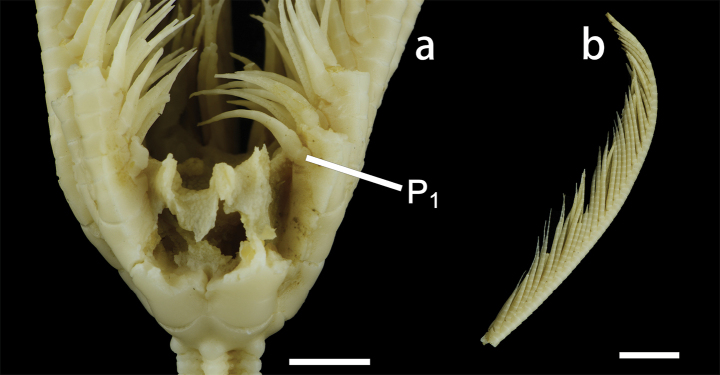
Endoxocrinus (Diplocrinus) kexuei sp. nov., holotype (MBM287584) **a** general view of crown **b** general view of pinnulars. P_1_: first pinnule. Scale bars: 5 mm (**a**); 1 cm (**b**).

**Figure 4.**
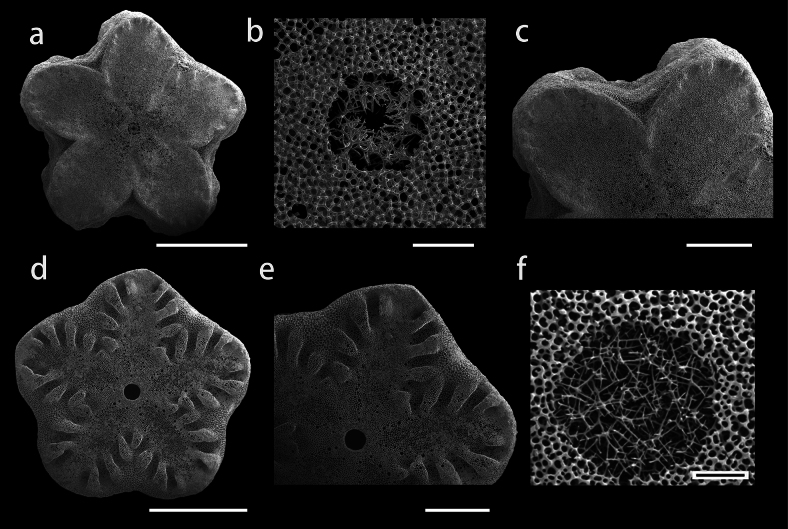
SEM views of stalk articulations in Endoxocrinus (Diplocrinus) kexuei sp. nov., holotype (MBM287584)(**a−e**); E. (Diplocrinus) alternicirrus (David et al. 2006, fig 20b) (**f**); cryptosymplexy (**a–c, f**) symplexy (**d–e**). **a** Distal nodal facet **b** and **f** close up on stereom within axial canal **c** petaloid and interpetaloid zone of cryptosymplexy **d** symplexy of an internodal’s facet **e** petaloid and interpetaloid zone of the symplexy. Scale bars: 1 mm (**a, c–e**); 100 µm (**b, f**).

**Figure 5.**
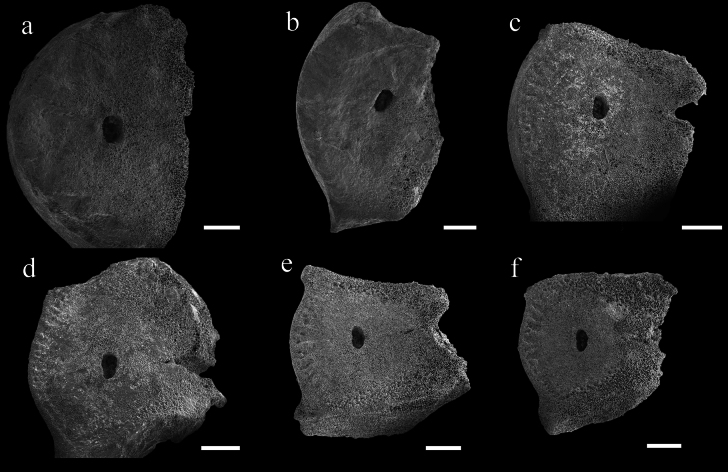
Endoxocrinus (Diplocrinus) kexuei sp. nov., holotype (MBM287584) **a** distal facet of IBr1 **b** proximal facet of IBr2 **c** distal facet of IIBr1 **d** proximal facet of IIBr2 **e** distal facet of IIIBr1 **f** proximal facet of IIIBr2. Scale bars: 600 µm.

**Figure 6.**
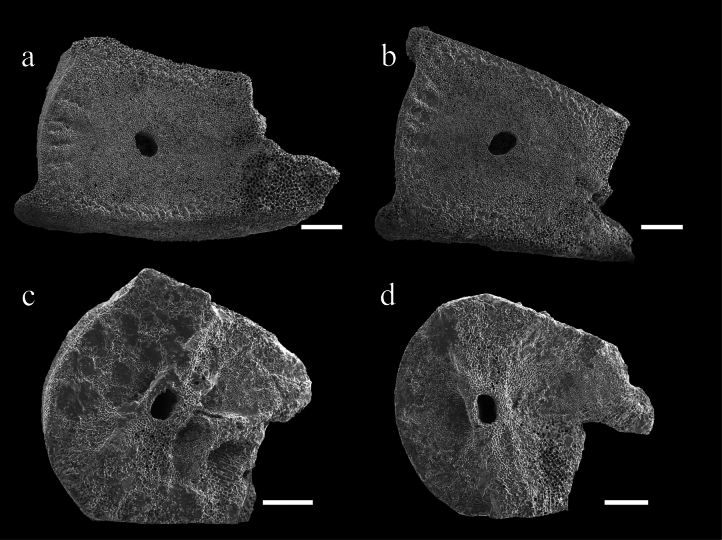
Endoxocrinus (Diplocrinus) kexuei sp. nov., holotype (MBM287584) **a** distal facet of br1 **b** proximal facet of br2 **c** distal facet of br5 **d** proximal facet of br6. Scale bars: 400 µm.

***Morphological description of the holotype*.** Specimen MBM287584 (Figs 1–6). Colour (Fig. 1a, b) pale yellow-pink in fresh samples, and white after alcohol immersion. Several arms and all cirri broken.

Arms 22, surface smooth, whole arm length 8–17cm; two or three divisions per ray, two of these rays have three divisions, rays closely aligned (Fig. 2a); all brachitaxes of two ossicles, united by non-muscular joints (synostosis). On all free arms, the first two brachials are connected by a synostosis muscular articulations present on the remaining branchials. IB_r1_ rectangular with distal lateral margins weakly everted. IB_r2_ pentagonal with lateral margin somewhat thickened and crenulated. IIB_r1+2_ and IIIB_r1+2_ with lateral flanges. IIB_r1_ and IIIB_r1_ with exterior lateral margin longer than interior. Proximal brachials (up tp B_r6_) approximately square, with varying degrees of crenulate on lateral margin, the edge regular, flat and close to straight, giving a very angular appearance to the brachials.

The non-functional ligamentary articulations IB_r1+2_, IIB_r1+2_ and IIIB_r1+2_, are clearly identified as synostosis. The synostosis of IB_r1+2_ very flat with synostosial stereom predominating and rectangular axial canal (Fig. 5a, b). However, at IIB_r1+2_ and IIIB_r1+2_ (Fig. 5c–f), while synostosial stereom predominats and, syzygial stereom forms rudimentary crenularium near the aboral edge of the facet; the syzygial stereom remains very irregular on the lateral edges.

On the free arms, non-muscular junctions are present only in B_r1+2_, whose general morphological features are similar to the secundibrachial or tertibrachials synostosis, with synostosial stereom forming a rudimentary crenularium on the aboral margin of the articular facet (Fig. 6a, b). The rest of the brachial articulations are typical isocrinid synarthries (Fig. 6c, d).

Basals form quadrilateral projections, protruding and separated from each other (W/L = 1.4–1.6). Radials hexagonal or pentagon, laterally adjacent (W/L = 2.4–2.8).

First pinnule (P_1_) on B_r2_ (Fig. 3a), with 9–11 pinnulars, approximately 8.1 mm long; second pinnular longest, up to 1.4 mm, penultimate segment slender, twice as long as wide. P_2_ to P_4_ short and wide; proximal segments relatively long, middle segments longest, and distal segments abruptly narrowed and shortened. P_10_ to P_15_ longest and slender, about 13.5–16.5 mm long, with 17–19 pinnulars; proximal segment wider, middle segments longer than wide (L/W = 1.4–1.6); distal pinnulars with small spines on dorsal edges. Pinnules gradually becoming shorter towards the distal of the arm. P_20_ to P_29_ composed of 6–15 segments, pinnulars longer than wide (L/W = 1.6–2.0), except the first pinnular.

Stout stalk about 7.8 cm long and counts 39 whole columnals, distal nodal slightly broken proximal stalk gradually turning from star-shaped to pentagonal in cross-section, 5 mm in diameter proximally and 4.3 mm distally, the longest noditaxis is the seventh (6.92 mm) with eight nodals, proximal nodals are 1.2 mm in height and distal ones being 2.1 mm tall in this noditaxis; less than 5 cirri on each nodal, usually two or three, four on the 6^th^ nodal and two on the 7^th^ nodal. Proximal cirri directed upward, forming an acute angle with the proximal stalk; rudimentary cirri on the first to third nodal. The number of interrnodal per noditaxis is 5 (Fig. 2b). Between two nodals, the internodal exactly in the middle is slightly higher than the others. Cirri remaining rudimentary, usually before the 3^th^ nodal. Fully developed cirrus slender with 30 cirrals, 6.3–8.5 cm long (Fig. 2c) (others incomplete or missing); cirrus sockets large and round, occupying approximately the entire height of nodal (Fig. 2b). The first 3 segments are relatively short. c_1_–c_4_ with W/L = 1.7–2.3; c_5_ to c_9_ longest, longest cirral with W/L = 0.8–1; subsequent cirrals gradually shorter, c_13_–c_15_ with W/L = 1.0–1.1; terminal claw quite small, slightly longer than the penultimate segment, no opposing spine; distalmost three cirri curved downward for fixation.

Nodal and infranodal united by cryptosymplexy. The cryptosymplexy (Fig. 4a) is pentalobate and flat, with synostosial stereom occupying most of it and syzygial stereom restricted to its outer margin. The round axial canal (Fig. 4b) is partially filled with a relatively long spicule network separated from the perilumen and preserving an irregular secondary lumen. Petaloid zones (Fig. 4c) pear-shaped, interpetaloid zone is dominated by syzygial stereom and without axial groove. Symplexies present between internodals (Fig. 4d), each petaloid zone having 6–8 crenulae and open lanceolate areola and interpetaloid zone (Fig. 4e) is dominated by labyrinthic stereom.

**Figure 7.**
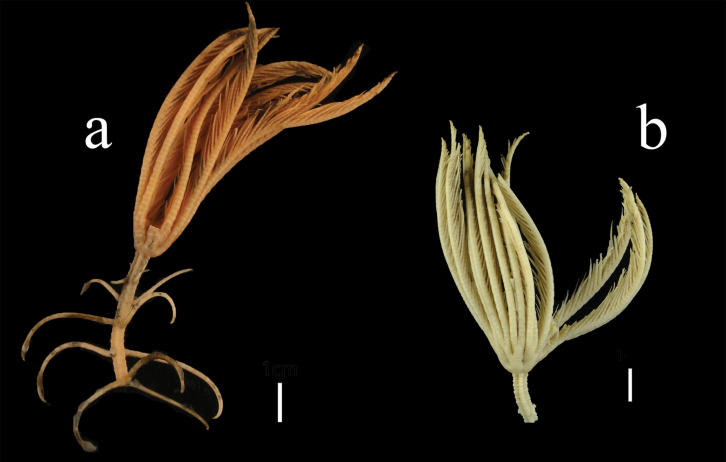
Endoxocrinus (Diplocrinus) kexuei sp. nov., paratype (MBM287585) **a** photograph of the entire organism taken directly after collection presenting the original colour **b** overall photo after preservation in alcohol. Scale bars: 1 cm.

**Figure 8.**
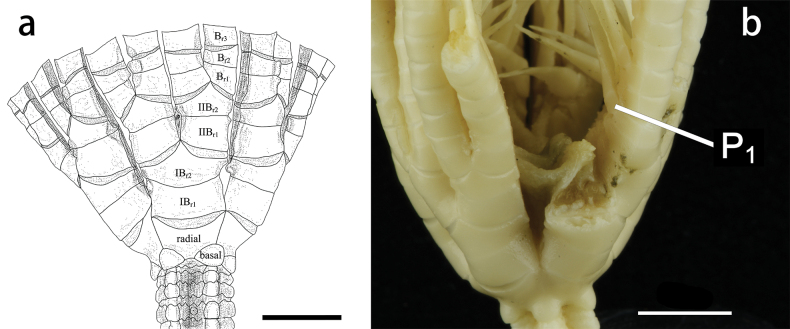
Endoxocrinus (Diplocrinus) kexuei sp. nov., paratype (MBM287585) **a** proximal stalk and base of crown **b** general view of crown, P_1_, first pinnule. Scale bars: 5 mm.

**Figure 9.**
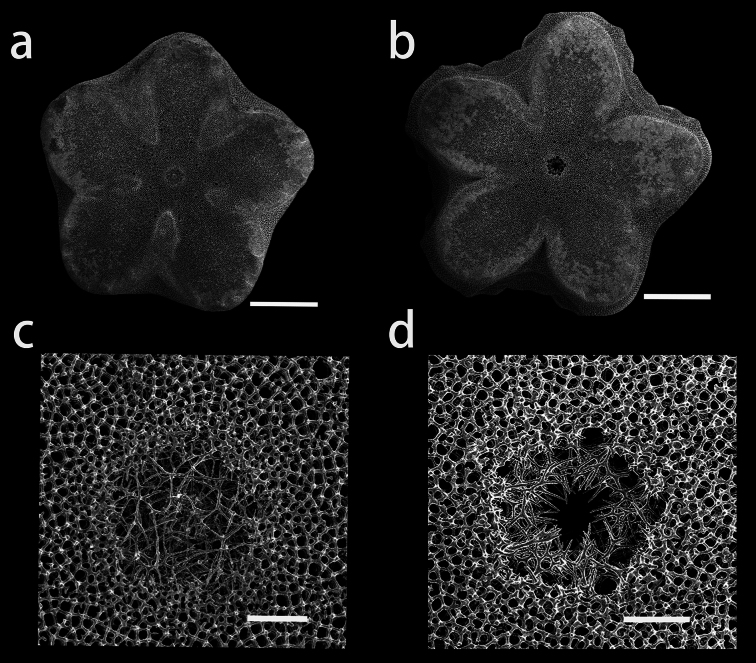
SEM views of cryptosymplexies in Endoxocrinus (Diplocrinus) kexuei sp. nov., paratype (MBM287585) **a–c** and **b–d** are from two different articulations **b–d** being more proximal **a** proximal infranodal facet **b** distal nodal facet **c** and **d** close-ups on the axial canal. Scale bars: 1 mm (**a, b**); 100 µm (**c, d**).

***Morphological description of the paratype*.** Specimen MBM287585 (Figs 7–9) incomplete, part of the stalk and all cirri are missing (Fig. 6b). Paratype is similar to the holotype, yet smaller. Differences are listed below.

20 smooth arms, up to 7–8 cm long. Two brachials per brachitaxes and two divisions per ray. IB_r1+2ax_ and IIB_r1+2ax_ (Fig. 8a). Proximal branchials with lateral edges less waved than in the holotype. Branchial surface smooth. IB_r1+2_, IIB_r1+2_ and IIIB_r1+2_, have small lateral flanges.

Basals rhombic, prominent and obviously apart from each other, W/L = 1.1–1.5. Radials hexagonal or pentagon, W/L = 1.9–2.3.

P1 on B_r2_ (Fig. 8b), with 8–9 pinnulars. Oral pinnule short; proximal pinnulars thick and wide, distal pinnulars become slender and longer. Middle pinnule longest, with 11–13 pinnulars, 13.0 mm long. At the end of arms, pinnule shorter or absent.

The remaining stalk length is about 1.7 cm, proximal column cross-section stellate to pentagonal.

Proximal infranodal columnal facet (Fig. 9a) with slight symmorphy in inner part, pear-shaped petaloid zones, and axial canal completely filled with dense thin stereom network without secondary lumen (Fig. 9c). Cryptosymplexies (Fig. 9b) relatively flat without marked symmorphy and with facets mainly covered by synostosial stereom. Outer margin zone and interpetaloid zone with syzygial stereom predominating. Axial canal (Fig. 9d) markedly pentagonal and partially filled with a relatively long spicule network separated from the perilumen and preserving an irregular secondary lumen.

Quantitative morphological characters are reported in Table 1.

##### Distribution.

Only known from the cold seep in the Taixinan Basin, South China Sea, at a depth of 833.7 m.

##### Remarks.

The genus *Endoxocrinus* now contains four extant species. According to David et al. (2006), the new species resembles mostly E. (Diplocrinus) alternicirrus in terms of arm division series, shape of the cross-section of stalk, and the number of internodal per mature noditaxis (Table 3). In E. (Diplocrinus) alternicirrus, the arms are up to 64 in number and 10–12 cm in length, while in E. (Diplocrinus) kexuei sp. nov., the arms are only 20–22 in number and are shorter (8–14 cm). The number of arms is variable among *Endoxocrinus* species (Table 3). The arm number is usually considered as an adaptive trait that often depends on depth, food supply and hydrodynamics (Roux 1987; David et al. 2006). In E. (Diplocrinus) kexuei sp. nov., the proximal cirri are directed upward and cirri are rudimentary until the 3^th^ nodal, differing from E. (Diplocrinus) alternicirrus, in which the proximal cirri are oriented downward and cirri are rudimentary until 5^th^ nodal. These characteristics are similar to E. (Diplocrinus) wyvillethomsoni, in which the proximal cirri are oriented upward and the rudimentary cirri are present to the 3^rd^ nodal.

**Table 3.** Comparison of the diagnoses of the *Endoxocrinus* species, summarized from David et al. (2006).

E. (Diplocrinus) kexuei sp. nov.	E. (Diplocrinus) alternicirrus	E. (Diplocrinus) maclearanus	E. (Diplocrinus) wyvillethomsoni	E. (Endoxocrinus) parrae
arms 20–22	arms up to 64	arms 14–30	arms 10–21	arms 23–58
arms smooth	arms smooth	arms smooth	arms smooth	arms smooth to serrated
arms 8–14 cm long	arms up to 15.3 mm long	arms up to 10 cm long	arms up to 10.5 cm long (mean 7 cm)	–
IB_r1+2_ synostosis very flat with synostosial stereom predominating	IB_r1+2_ synostosis relatively flat with synostosial stereom predominating, small syzygial crenulation near aboral edge of facet	proximal synostoses at B_r1+2_ relatively flat with syzygial stereom irregularly developed	non-muscular articulation B_r1+2_ intermediate between synostosis and syzygy, showing a general symmorphy, tending to a true synostosis distally and to a syzygy between primibrachials	–
stalk axial canal rectangular	stalk axial canal rectangular	stalk axial lumen rectangular	axial canal lumen bilobate	–
stalk length about 6.5 cm long	stalk length variable up to 14 cm	stalk <9 cm long (mean 3.8 cm)	stalk length strongly variable, 3.5–22.5 cm	–
internodals per mature noditaxis 5–8	internodals per mature noditaxis 4–10 usually 5–6	internodals per mature noditaxis usually 6–14	internodals per mature noditaxis 20–56 (mode 32)	internodals per mature noditaxis usually 6–14 (mode in local populations 8–12)
stalk stellate to pentagonal cross-section	stalk stellate to pentagonal cross-section	stalk pentalobate to pentagonal cross-section	middle and distal stalk pentagonal to circular in cross-section	stalk in adult pentalobate to pentagonal in cross-section
prox stalk diameter 5 mm	prox stalk diameter 4–6 mm up to 7.9 mm	prox stalk diameter up to 5.1 mm	prox diameter of stalk up to 4.2 mm (mean 2.9 cm)	prox diameter of stalk usually 4–5 mm
less than 5 cirri per nodal	less than 5 cirri per nodal	cirri usually 5 per nodal	always 5 robust cirri per nodal	usually 5 cirri per nodal
Proximal cirri directed upward	proximal cirri oriented downward	cirri oriented upward	proximal cirri oriented upward	–
cirrus sockets large and round	cirri socket nearly circular	–	–	–
The first 3 segments are relatively short, c5 to c9 longest, subsequent cirrals gradually shorter	cirrals long	–	–	–
up to 30 robust cirrals per cirrus	up to 35 cirrals per cirrus (usually 28–30)	–	–	with up to 44 cirrals per cirrus (usually >30)
cirri rudimentary until 3^th^ nodal	cirri rudimentary until 5^th^ nodal	–	rudimentary cirri present to 3^rd^ nodal	–
cryptosymplexies without marked symmorphy and with facets mainly covered by synostosial stereom	interpetaloid zone of cryptosymplexies with syzygial stereom predominating but with strong symmorphy in inner portion	cryptosymplexies with undulating symmorphic surface, synostosial stereom predominating on interpetaloid zones	cryptosymplexies flat or with slight general symmorphy with syzygial stereom predominating on interpetaloid zones and on a regular outer border of the facet	cryptosymplexies flat or with a slight symmorphy of inner interpetaloid zone, with syzygial stereom predominating in interpetaloid zones
axial canal usually incompletely filled with stereom needlelike network, separated from perilumen	axial canal with long thin spicules, distinct from perilumen	axial canal filled with short thick spicules clearly separated from perilumen	axial canal filled up by large meshed stereom not clearly separated from perilumen	axial canal filled up by an irregular and variable network not clearly separated from perilumen
preserving an irregular secondary lumen	no secondary lumen	secondary lumen small or absent	secondary lumen small or absent	a small to large secondary lumen
Western Pacific	Western Pacific	Eastern Brazil, Bahamian	South European Atlantic Shelf, Eastern Caribbean	Gulf of Mexico and Caribbean Sea
833.7 m	600–2000 m	200–300 m	1000–3000 m	150–2000 m

Cryptosymplexies exhibit traits that are more relevant at the species level. In E. (Diplocrinus) kexuei sp. nov., the cryptosymplexies present no marked symmorphy with facets mainly covered by synostosial stereom, while in E. (Diplocrinus) alternicirrus, the interpetaloid zone of cryptosymplexies with syzygial stereom predominating but with strong symmorphy in inner portion. In E. (Diplocrinus) wyvillethomsoni and E. (Endoxocrinus) parrae, cryptosymplexies are flat or with slight symmorphy with syzygial stereom predominating on interpetaloid zones. In E. (Diplocrinus) maclearanus, the cryptosymplexies with undulating symmorphic surface, synostosial stereom predominating on interpetaloid zones. The difference in the characteristics of stalk cryptosymplexy supports our classification at the species level rather than at the subspecies level, in accordance with Roux (1977) and David et al. (2006). The axial canal structures show distinct differences among *Endoxocrinus* species. In E. (Diplocrinus) kexuei sp. nov., the axial canals are incompletely filled with long spicules, separated from the perilumen and preserve an irregular secondary lumen, while in E. (Diplocrinus) alternicirrus, the axial canals are completely filled with a dense mesh of long thin spicules without secondary lumen (Fig. 4f) (Roux 1977, David et al. 2006). In E. (Diplocrinus) maclearanus, E. (Endoxocrinus) parrae and E. (Diplocrinus) wyvillethomsoni, the axial canal is filled with short thick spicules, large meshed stereom, and irregular network, respectively. In E. (Diplocrinus) maclearanus and E. (Endoxocrinus) parrae, the secondary lumen is small or absent. Endoxocrinus (Endoxocrinus) parrae has a small to large secondary lumen.

### ﻿Key to the genus *Endoxocrinus*

**Table:** 

1	IIIB_r_ and following series of three ossicles, developed exteriorly	**Endoxocrinus (Endoxocrinus) parrae (Gervais in Guérin, 1835)**
–	IIIB_r_ and following series, when present, of two ossicles only	**2**
2	Internodes long, usually of 20–56 columnals (mode 32)	**Endoxocrinus (Diplocrinus) wyvillethomsoni (Thomson, 1872)**
–	Internodes fewer columnals, usually of 5–14	**3**
3	Cirri usually 5 per nodal	**Endoxocrinus (Diplocrinus) maclearanus (Thomson, 1878)**
–	Less than 5 cirri per nodal	**4**
4	Preserving an irregular secondary lumen	**Endoxocrinus (Diplocrinus) kexuei sp. nov.**
–	No secondary lumen	**Endoxocrinus (Diplocrinus) alternicirrus (Carpenter, 182)**

### ﻿Barcoding, phylogenetic relationships, and taxonomic implication

**Figure 10.**
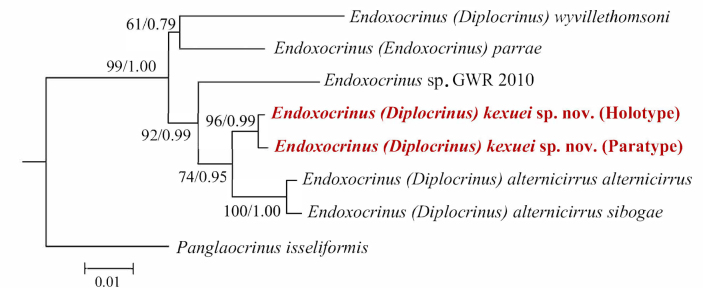
Maximum-likelihood (ML) tree of *Endoxocrinus* using combined sequences of *COI* (619 bp), *16S* (328 bp) and *28S* (736 bp). As the topologies of ML or BI are congruent, the ML tree was used to represent the phylogeny. The number at each node represents bootstrap values (BP) (left) and Bayesian posterior probability (BPP) (right). The new species is highlighted in red.

The interspecific genetic distance (K2P) within the genus *Endoxocrinus* was calculated based on the *COI* gene. The interspecific distances within *Endoxocrinus* ranged from 2.1% to 8.5%. The genetic distance within the new species is 0.5%. Endoxocrinus (Diplocrinus) kexuei sp. nov. showed the shortest genetic distance with E. (Diplocrinus) alternicirrus (2.1–2.5%) and the greatest distance with E. (Diplocrinus) wyvillethomsoni (7.9%) (Table 4). The phylogenetic tree of extant *Endoxocrinus* derived from BI and ML analyses showed consistent results (Fig. 10). Endoxocrinus (Diplocrinus) kexuei sp. nov. is nested within *Endoxocrinus* and is sister clade to E. (Diplocrinus) alternicirrus. Both the genetic distance and phylogenetic relationship are consistent with the classification based on morphological evidence. E. (Diplocrinus) kexuei sp. nov. is similar to its congener E. (Diplocrinus) alternicirrus, with the same division series, cross-section of stalk, and the same number of internodals per mature noditaxis.

**Table 4.** The genetic distance of *COI* gene (K2P) within *Endoxocrinus* species.

	1	2	3	4	5	6	7
1 E. (Diplocrinus) kexuei sp. nov. (holotype)							
2 E. (Diplocrinus) kexuei sp. nov. (paratype)	0.005						
3 E. (Diplocrinus) wyvillethomsoni	0.079	0.081					
4 E. (Endoxocrinus) parrae	0.060	0.062	0.078				
5 E. (Diplocrinus) alternicirrus alternicirrus	0.025	0.030	0.085	0.069			
6 E. (Diplocrinus) alternicirrus sibogae	0.021	0.026	0.081	0.069	0.006		
7 *Endoxocrinus* sp. GWR-2010	0.042	0.047	0.074	0.061	0.051	0.047	

## ﻿Conclusion

Based on morphology and phylogenetic analysis, one *Endoxocrinus* species is recognized and described as new to science in this study: Endoxocrinus (Diplocrinus) kexuei sp. nov. The new species is similar to E. (Diplocrinus) alternicirrus in external morphology, but differs by showing cryptosymplexies without marked symmorphy, and an axial canal usually incompletely filled with a lattice needlelike network, preserving an irregular secondary lumen. The phylogenetic analysis provided support for the establishment of the new species. The genetic distances between the new species and E. (Diplocrinus) alternicirrus are the smallest. Endoxocrinus (Diplocrinus) kexuei sp. nov. is the first extant stalked crinoid discovered in a cold seep area in the South China Sea.

## Supplementary Material

XML Treatment for Diplocrinus

XML Treatment for Endoxocrinus (Diplocrinus) kexuei

## ﻿Additional information

### Author ORCIDs

Shao'e Sun https://orcid.org/0009-0003-5774-6209

Zijie Mei https://orcid.org/0009-0002-6818-0565

Zhongli Sha https://orcid.org/0000-0002-2192-3758

### Data availability

In accordance with FAIR principles, the *COI*, *16S* and *28S* sequences in this study (Suppl. material 1) can be found in the Supplemental Materials and openly available in GenBank.

## Supplementary materials

Supplementary material 1 Six sequence datasets from two specimens Data type txt Explanation note Sequence information needed to construct phylogenetic trees. https://binary.pensoft.net/file/1351511This dataset is made available under the Open Database License (http://opendatacommons.org/licenses/odbl/1.0/). The Open Database License (ODbL) is a license agreement intended to allow users to freely share, modify, and use this Dataset while maintaining this same freedom for others, provided that the original source and author(s) are credited.Shao’e Sun, Zijie Mei, Zhongli Sha
